# Studying microbial triglyceride production from corn stover saccharides unveils insights into the galactose metabolism of *Ustilago maydis*

**DOI:** 10.1186/s12934-024-02483-1

**Published:** 2024-07-20

**Authors:** Paul Richter, Jathurshan Panchalingam, Katharina Miebach, Kerstin Schipper, Michael Feldbrügge, Marcel Mann

**Affiliations:** 1 Aachener Verfahrenstechnik – Chair of Biochemical Engineering, RWTH Aachen University, 52074 Aachen, Germany; 2 Institute for Microbiology, Heinrich Heine University Düsseldorf, 40225 Düsseldorf, Germany; 3 Bioeconomy Science Center (BioSC), 52425 Jülich, Germany

## Abstract

The global demand for plant oil has reached unprecedented levels and is relevant in all industrial sectors. Driven by the growing awareness for environmental issues of traditional plant oils and the need for eco-friendly alternatives, microbial oil emerges as a promising product with significant potential. Harnessing the capabilities of oleaginous microorganisms is an innovative approach for achieving sustainable oil production. To increase economic feasibility, it is crucial to explore feedstocks such as agricultural waste streams as renewable resource for microbial bioprocesses. The fungal model *Ustilago maydis* is one promising organism in the field of microbial triglyceride production. It has the ability to metabolize a wide variety of carbon sources for cell growth and accumulates high amounts of triglycerides intracellularly. In this study we asked whether this large variety of usable carbon sources can also be utilized for triglyceride production, using corn stover saccharides as a showcase.

Our experiments revealed metabolization of the major saccharide building blocks present in corn stover, demonstrating the remarkable potential of *U. maydis*. The microorganism exhibited the capacity to synthesize triglycerides using the saccharides glucose, fructose, sucrose, xylose, arabinose, and galactose as carbon source. Notably, while galactose has been formerly considered as toxic to *U. maydis*, we found that the fungus can metabolize this saccharide, albeit with an extended lag phase of around 100 hours. We identified two distinct methods to significantly reduce or even prevent this lag phase, challenging previous assumptions and expanding the understanding of *U. maydis* metabolism.

Our findings suggest that the two tested methods can prevent long lag phases on feedstocks with high galactose content and that *U. maydis* can produce microbial triglycerides very efficiently on many different carbon sources. Looking forward, exploring the metabolic capabilities of *U. maydis* on additional polymeric components of corn stover and beyond holds promise for innovative applications, marking a significant step toward environmentally sustainable bioprocessing technologies.

**Supplementary Information:**

The online version contains supplementary material available at 10.1186/s12934-024-02483-1.

## Background

Plant oils are indispensable in today’s industry. As a flavor carrier in food, as an additive in cosmetics, in the form of emulsions in paints and detergents or as a blend in fuel production - vegetable oils are utilized in a vast range of consumer products [1]. Due to its variable utilization properties and a rising world population, the demand for plant oils has continuously increased in the last years [2] and is expected to grow even further [3]. This effect will also be exacerbated by potential energy crises, which are increasingly likely due to climate change [4, 5]. Although plant oils are considered a renewable raw material, they have a justifiably poor reputation due to the high production volumes required and the associated monocultures and deforestation of rainforests. The most relevant plant utilized for plant oil production is the oil palm and the derived palm oil [6]. Another critical example is rapeseed oil, which is directly competing with food production, because of its utilization in biofuels and because of land use [7].

Microbial triglyceride production with established oleaginous microorganisms such as *Yarrowia lipolytica* or *Rhodosporidium toruloides* represents a promising alternative to conventional plant oil production [8]. We here introduce *Ustilago maydis* as a novel prime candidate for microbial oil production [9]. This fungal model belongs to the phylum of the basidiomycetes and is known for causing corn smut disease, which is typically resulting in the formation of tumors in which fungal spores develop. Remarkably, these tumors are traditionally relished a delicacy in central and south America, supporting the notion that it is not dangerous for humans. Besides its role as a fungal model in basic biology, *U. maydis* also offers great potential for various biotechnological applications [10]. The main advantage of using *U. maydis* over other oleaginous microorganisms lies in its versatile genetic potential, which enables efficient metabolic engineering and optimization for various industrial processes. Furthermore, this versatility can be used in processes to convert as many carbon sources as possible into the desired target product. In the context of this paper, this potential was used to produce a strain that is able to store triglycerides intracellularly and can metabolize a variety of different carbon sources. In order to limit the product to triglycerides, potential synthesis pathways of two by-products were knocked out.

The oil production of this *U. maydis* strain MB215Δcyp1Δemt1 and in most other oleaginous organisms is induced by a secondary substrate limitation, commonly nitrogen. Therefore, it can easily be initiated via process control [11]. Microbial triglycerides offer several advantages over conventional plant oils. For instance, it is possible to adapt media compositions or implement genetic modifications to tailor the process towards the desired application [12]. In addition, land use for oil production could be reduced substantially and renewable raw materials or waste streams could be utilized as feedstock for the production process [13]. This could create a sustainable alternative to conventional plant oils without competing directly with food production.

Plant raw materials and plant-based agricultural waste streams consist of complex combinations of various carbon sources available for potential utilization. Besides the main components of plant cell walls cellulose and lignin, different hemicelluloses and soluble sugars are present in plant biomass [14, 15]. The proportions vary widely depending on the plant species and the thereof resulting waste stream. Since lignocellulosic feedstock became interesting for the biotechnological industry, various methods have been published to break down the polymeric components of plant biomass, especially cellulose and hemicellulose, into individual saccharide building blocks. Most plant waste streams consist mainly of the basic building blocks xylose, glucose, arabinose, galactose, mannose, and fructose [15]. In addition, plant biomass and the associated hydrolysates often contain larger amounts of acetate ranging from 2 to 5% (w/w) [15, 16]. Depending on the organism used for biomass conversion, the individual components of plant biomass often have positive or negative effects on the process.

In this study, we aim to address several key aspects of microbial triglyceride production using *U. maydis*, particularly focusing on the metabolization of the individual pentose and hexose building blocks found in plant biomass. Special emphasis is put on galactose, as it has been postulated to inhibit the growth of different *U. maydis* strains and other yeasts due to the formation of toxic intermediates such as galactose-1-phosphate during metabolization [17–19]. The formation of galactose-1-phosphate depends on the metabolic pathway used and the expressed enzyme levels of the pathway [20]. The Leloir pathway, found in nearly all eukaryotes, stands out as the most prevalent and evolutionarily preserved route, featuring galactose-1-phosphate as a key intermediate [21]. However, recent studies show that organisms that can metabolize galactose, like many filamentous fungi such as *Aspergillus niger* or *Trichoderma reesei*, show some unique genetic characteristics [22, 23]. For example, the genes of the Leloir pathway are not arranged in clusters, as in yeasts like *S. cerevisiae*, which allows a much more versatile regulation of the metabolic pathway [20]. In addition, filamentous fungi have at least one additional degradation pathway for galactose, the oxidoreductive pathway [24]. The oxidoreductive pathway enables the degradation of galactose without the accumulation of the toxic intermediate galactose-1-phosphate. Although galactose has been reported to show toxic effects on *U. maydis*, there is genetic evidence for the possibility of galactose metabolization [25–27]: Interestingly the genome of *U. maydis* contains both genes encoding enzyme for the Leloir-pathway, but also genes for enzymes that belong to the oxidoreductive pathway [21]. Although the Leloir pathway is the active one under standard cultivation conditions [27, 28], there is also evidence that genes of the oxidoreductive metabolic pathway are connected to filamentous growth [28] and often associated with external stress factors [29]. For the established oleaginous yeast *Yarrowia lipolytica*, the utilization of galactose could be demonstrated, although similar metabolic barriers should exist as with *U. maydis* [30].

The purpose of this study is to investigate the potential of *U. maydis* for galactose assimilation and triglyceride production, and to compare its performance with other sugars under various conditions. To achieve this, we focused on the following objectives: (1) evaluating the physiological and morphological responses of *U. maydis* to galactose as a carbon source, (2) assessing the oxygen transfer rate during cultivation, and (3) comparing the efficiency of galactose assimilation with other sugars. Additionally, we explored methods to shorten the lag phase on galactose by exploiting the history-dependent behavior of carbon source adaptation and the metabolic properties of glucose and galactose mixtures.

## Materials and methods

### Microorganism

The organism utilized for the experiments was *Ustilago maydis* MB215Δcyp1Δemt1, deposited at DSM17147 as MB215cyp1emt1 [31]. *U. maydis* MB215Δcyp1Δemt1 was genetically modified to eliminate both the synthesis of ustilagic acid and mannosylerythritol lipids by deletion of the genes *cyp1* and *emt1* encoding the enzymes for central catalytic steps in the two glycolipid biosynthesis pathways. The strain shows reduced byproduct formation and a high concentration of intracellularly accumulated triglycerides. The strain reaches high titer production due to its robust lipid biosynthesis pathways, yeast-like growth facilitating easy cultivation, and inducibility of lipid biosynthesis pathway with nitrogen limitation. The strain was stored in a 9 g∙L^− 1^ sodium chloride solution with a glycerol concentration of 200 g∙L^− 1^ at -80 °C.

### Media composition

For all cultivations, a modified Verduyn mineral medium [32] was used with the following composition, produced, if not stated otherwise, by Carl Roth GmbH + Co. KG (Karlsruhe, Germany): 1.6 g∙L^− 1^ (NH_4_)_2_SO_4_, 0.5 g∙L^− 1^ KH_2_PO_4_, 0.2 g∙L^− 1^ MgSO_4_ ∙ 7H_2_O, 0.01 g∙L^− 1^ FeCl_3_ ∙ 6H_2_O and 1 mL∙L^− 1^ trace element solution, which contained: 15 g∙L^− 1^ EDTA, 3 g∙L^− 1^ FeSO_4_· 7H_2_O, 0.84 g∙L^− 1^ MnCl_2_ · 2H_2_O, 4.5 g∙L^− 1^ ZnSO_4_ · 7H_2_O, 0.3 g∙L^− 1^ CuSO_4_ · 5H_2_O, 0.3 g∙L^− 1^ CoCl_2_ · 6H_2_O, 0.4 g∙L^− 1^ Na_2_MoO_4_ · 2H_2_O, 4.5 g∙L^− 1^ CaCl_2_· 2H_2_O, 1 g∙L^− 1^ H_3_BO_3_ and 0.1 g∙L^− 1^ KI. The respective carbon source was supplemented, if not stated otherwise, to a final concentration of 100 g∙L^− 1^ glucose equivalents normalized with the molar amount of carbon atoms. For all experiments involving galactose, D(+)-galactose from AppliChem GmbH, Germany was used. Due to a strong acidification of the culture while producing triglycerides, the medium was buffered with 0.4 M 2-(N-Morpholino)-ethane sulfonic acid (MES) buffer, which was adjusted to a starting pH value of 6.5. The different stock solutions of ammonium sulfate, potassium dihydrogen phosphate, magnesium sulfate, iron(III) chloride, trace element solution, and the different carbon sources were sterilized by filtration with a 0.2 μm cut-off filter and supplemented directly before cultivation. For the pre cultures an adapted media composition of the Verduyn medium was used, in which the carbon source concentration was adjusted to 20 g∙L^− 1^ glucose equivalent and the (NH_4_)_2_SO_4_ concentration was adjusted to 5 g∙L^− 1^.

### Cultivation conditions

The cultivations were performed in an inhouse built Respiratory Activity Monitoring System (RAMOS) [33]. Detailed information about the setup and technology can be found in Anderlei et al. 2001 and 2004 [33, 34]. For the cultivation, 250 mL RAMOS shake flasks were filled with 20 mL of medium, inoculated with an initial optical density at 600 nm of 0.1 from a pre culture. Before inoculation, the pre culture was washed with 9 g∙L^− 1^ sodium chloride solution to avoid transferring media components into the main cultivation. The cultivation was performed in a tempered shaker (Kuhner Shaker GmbH, Herzogenrath, Germany) at 30 °C. For a sufficient oxygen supply, the shake flasks were shaken at 350 rpm, with a shaking diameter of 50 mm. For the pre cultures the same cultivation conditions were used, but the inoculation was done with an initial optical density at 600 nm of 0.1 from a cryo culture.

### Offline analytics

Quantification of the carbon sources glucose, fructose, sucrose, arabinose, xylose, and galactose was carried out via high-performance liquid chromatography (HPLC). The analyzed data was used to calculate the product yields in grams of produced oil per gram of consumed carbon source. HPLC measurements were performed in the Prominence HPLC system (Shimadzu, Duisburg, Germany). The HPLC system was equipped with the following columns and detectors: precolumn Organic Acid Resin (40 × 8 mm, CS-Chromatography, Service, Langerwehe, Germany), column Organic Acid Resin (250 × 8 mm, CS-Chromatography Services, Langerwehe, Germany), detector RID-20 A Refraktometer (Shimadzu, Duisburg, Germany). The mobile phase consisted of 5 mM H_2_SO_4_. The flow rate was adjusted to 0.8 mL∙min^− 1^ at 35 °C and an injection volume of 20 µL was used. For HPLC sample preparation, the cultivation broth was centrifuged for 10 min at 15,093 rcf and subsequently filtered with a 0.2 μm cut-off filter.

For triglyceride quantification 2.4 mL of the culture broth was sonicated with the Fisherbrand™ Model 120 Sonic Dismembrator with a 1/8’’ Microtip (Fisher scientific, Schwerte, Germany) to disrupt the cells. 2 mL of the crude cell extract was used in an extraction process according to Matyash *et al*. 2008 with 5 mL methyl-tert-butyl ether (MTBE) and 1.5 mL methanol [35]. After phase separation, the solvent phase was collected in dried weighed glass vials and evaporated at room temperature. After the evaporation, the glass vials were weighed again until weight consistency.

The Nikon Eclipse E600 POL (Nikon Corporation Industrial Metrology Business Unit, Tokyo, Japan) was used for recording the microscopic images of the culture broths. The microscope was equipped with the Dark Low Low contrast (DLL) objectives 10x/0.25 and 100x/1.3 oil. When using the objective with a tenfold magnification, the phase contrast annulus Ph1 (Nikon Corporation Industrial Metrology Business Unit, Tokyo, Japan) was selected. The phase contrast annulus Ph3 (Nikon Corporation Industrial Metrology Business Unit, Tokyo, Japan) was selected for using the objective with a hundredfold magnification. In addition, immersion oil was used with the 100x/1.3 oil objective.

## Results and discussion

### Influence of carbon source on triglyceride production

**Fig. 1:**
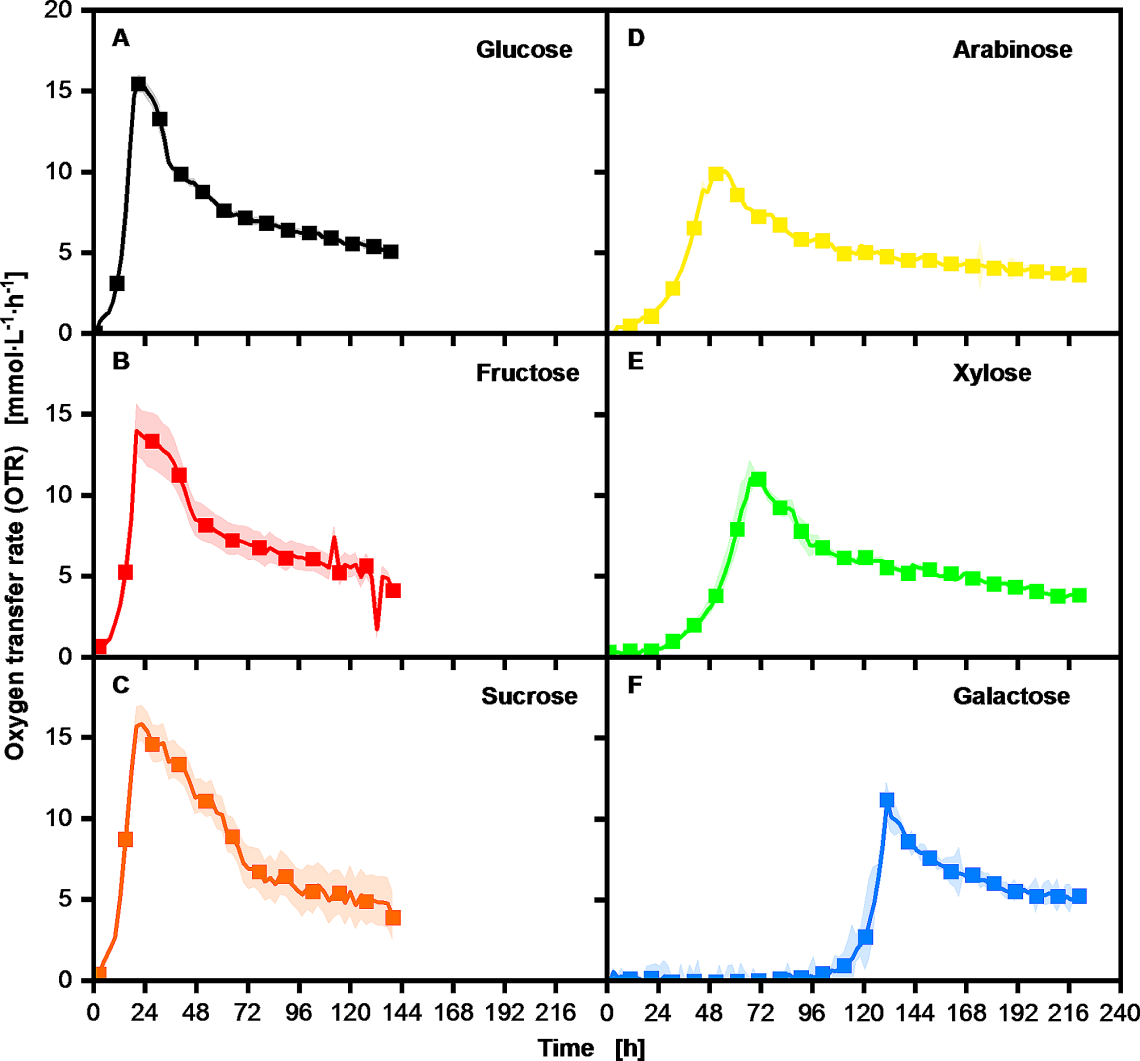
Comparison of the respiration activity of *U. maydis* MB215Δcyp1Δemt1 cultivations on different carbon sources. The Verduyn medium was supplemented with 100 g∙L^− 1^ glucose equivalent of the respective carbon source. Cultivations were conducted in duplicate, with the average values represented as a line and the min/max values illustrated as error shadows. Carbon source consumption and additional recorded data are shown in the supplementary data S1

The growth and the triglyceride production of *U. maydis* on different saccharide building blocks of plant material were investigated first. Growth of *U. maydis* MB215Δcyp1Δemt1 was tracked using the respiratory activity of the organism measured by the RAMOS device. The respiratory activity of the organism on the different saccharides is represented in Fig. 1A - F by the oxygen transfer rate (OTR). In Fig. 1A, the reference cultivation on glucose as carbon source is shown. With this reference cultivation, the schematics behind triglyceride production can be described. The OTR starts to rise directly in the beginning of cultivation leading to an exponential growth phase. The exponential growth phase stops at around 20 h when nitrogen is depleted. This depletion is indicated by a peak formation in the OTR. After this first peak, a transition phase starts, where *U. maydis* adapts its biomass composition to the new condition under nitrogen limitation as previously shown by Klement et al. (2012) [36]. After this transition phase, no more biomass is generated. Instead, due to nitrogen limitation, the organisms start to accumulate triglycerides intracellularly until carbon source depletion or stop of the experiment.

The first two tested carbon sources, fructose (B) and the disaccharide sucrose (C), show only slight differences to the reference cultivation on glucose in the respiration activity. During cultivation using the pentoses arabinose (D) and xylose (E) as the sole carbon sources, a lag phase of 12 and 24 h, respectively, is observed. The cultivation on galactose (F) is particularly noteworthy. A lag phase of approximately 100 h was observed. This is significant in two aspects: firstly, it is surprising that growth occurs at all after the culture has been incubated for 100 h without detectable proliferation and secondly, growth on galactose appears to be feasible, contrary to previous findings of an earlier study, with a different experimental set up [21]. In order to prove that there was really no metabolic activity in the first 100 h of culture, we also plotted the carbon dioxide transfer rate (CTR) in the supplementary data (Figure S2) for the cultivation on galactose. Since no oxygen was consumed and no carbon dioxide was produced, we can rule out aerobic or anaerobic metabolism in the first 100 h of the cultivation. After the lag phase (if present), the exponential growth phase starts in all cultivations, indicating that upon adaptation growth is possible on all tested carbon sources. This aligns closely with the literature for growth of *U. maydis* on sucrose, fructose, arabinose, and xylose [37, 38]. To allow direct comparison, the maximum specific growth rate coefficient (µ_max_) on all carbon sources was deduced from the maximum exponential slope of the OTR [39]:

1 $$\:{\mu\:}_{max}=\:\frac{lnOTR\left(t\right)-lnOTR\left({t}_{0}\right)}{t-{t}_{0}}$$

**Fig. 2:**
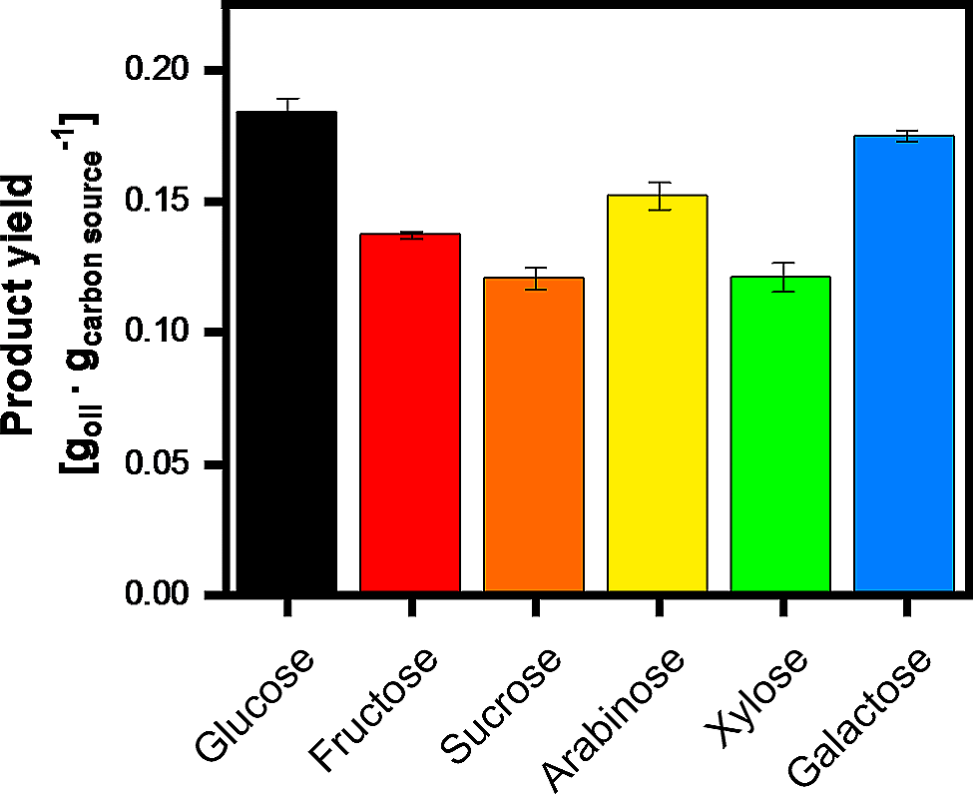
Product yields of triglycerides produced by *U. maydis* MB215Δcyp1Δemt1 on different carbon sources (relating to Fig. 1). Extraction was performed in biological replicates, with the average values represented in the column chart and the min/max values illustrated as error bar. The Product yield was calculated by dividing the oil concentration at the end of the cultivation by the used carbon concentration. Residual, non-consumed carbon source was determined by HPLC and included in the calculation by subtracting it from the initial used carbon source concentration. It should be mentioned here that the carbon source concentration initially used was calculated to a glucose equivalent. Carbon source consumption and additional recorded data is shown in the supplementary data S2

**Fig. 3:**
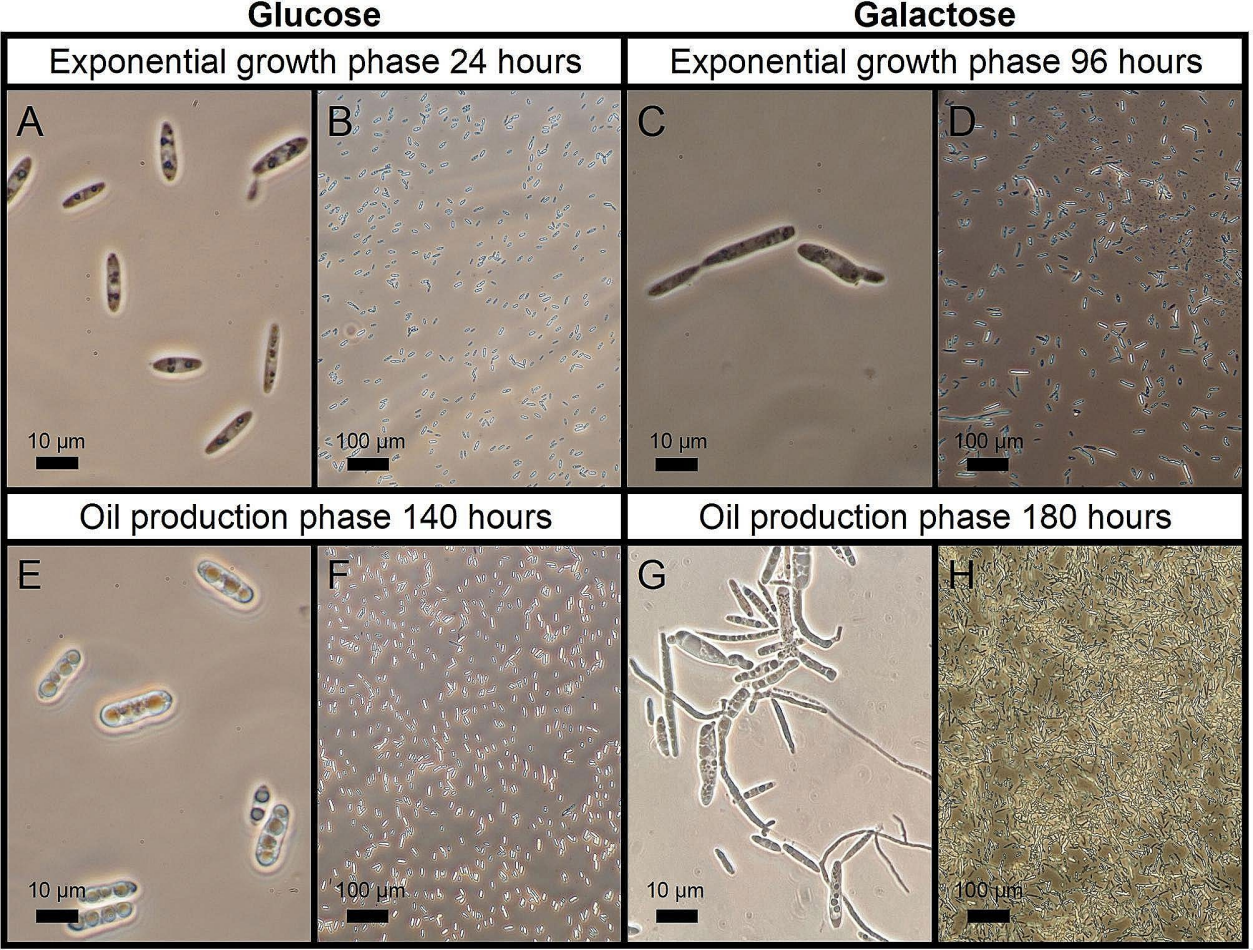
Microscopic images of *U. maydis* MB215Δcyp1Δemt1 cells grown on glucose and galactose as single carbon sources (relating to Fig. 1). Different sampling times resulting from a variation in the duration of the cultures lag phases. When using the objective with a tenfold magnification (**B, D, F, H**), the phase contrast annulus Ph1 (Nikon Corporation Industrial Metrology Business Unit, Tokyo, Japan) was selected. The phase contrast annulus Ph3 (Nikon Corporation Industrial Metrology Business Unit, Tokyo, Japan) was selected for using the objective with a hundredfold magnification (**A, C, E, G**). In addition, immersion oil was used with the 100x/1.3 oil objective

A graphical representation and a table of the calculated values are shown in the supplementary data (S1, S3). Comparable growth rates with 0.208 h^− 1^ for glucose, 0.190 h^− 1^ for fructose and 0.198 h^− 1^ for sucrose were observable. The pentoses arabinose and xylose show a more gradual incline, which is confirmed by a calculated growth rate of 0.070 h^− 1^ for arabinose and 0.087 h^− 1^ for xylose. The cultivation on galactose shows an intermediate growth rate of 0.145 h^− 1^ which, despite the long lag phase, is nearly comparable to the preferred carbon sources. The recorded values match the published values of 0.1 to 0.18 h^− 1^ on pure glucose [25, 40], while for the other carbon sources, to our knowledge no literature values are available to date. The residual concentrations of the different carbon sources were determined at the end of the cultivation by HPLC measurement. Significant residual saccharides were only detected for galactose, which however can be explained by the late growth initiation and the shorter lipid accumulation phase until end of the experiment compared to the other cultivations shown in Fig. 1. Evidence for the total galactose metabolization and conversion into oil is presented in one of the later cultivations (Fig. 2). To evaluate not only the growth of *U. maydis* on different carbon sources but also the productivity, the produced triglycerides were extracted and the concentrations between the different cultivations were compared. With the triglyceride concentrations and the amount of consumed carbon source a yield of triglyceride per consumed carbon was calculated and depicted in Fig. 3.

When comparing the productivity and product yields on the different carbon sources, it can be concluded that triglyceride production is possible on all tested carbon sources, although there are some variations in the specific product yield. Glucose and galactose show the highest product yields, with a yield of 0.18 ± 0.005 g_triglycerides_∙g_carbon source_^−1^ for glucose and a similar yield of 0.18 ± 0.002 g_triglycerides_∙g_carbon source_^−1^ for galactose, despite the much longer lag phase. A slightly lower product yield could be observed with the other tested saccharides sucrose, fructose, arabinose, and xylose. *Cianchetta et al.* postulated in 2023 a maximum theoretical product yield for other oleaginous yeasts of 0.31 g_triglycerides_∙g_carbon source_^−1^ on glucose but mentioned that documented conversion yields above 0.2 g_triglycerides_∙g_carbon source_^−1^ are rare [41]. Hence, the product yields on the different carbon sources gathered in this study agree well with the literature for other organisms on glucose and other carbon sources [41, 42]. There are also many articles based on renewable raw materials, like corn stover, as complex substrates, which exhibit a similar yield range from 0.18 to 0.2 g_triglycerides_∙g_carbon source_^−1^ [43–46]. A detailed compilation of several organisms and the specific product yields on different carbon sources is provided in the review article published in 2023 by Gallego-Garcia et al. [47]. In essence, the results clearly indicate the potential of *U. maydis* MB215Δcyp1Δemt1 for oil production, by its ability to metabolize a variety of different carbon sources. Importantly, the gained yields are similar to well-known microbial oil producers without intensive process optimization. At the same time, the results raise curiosity about galactose metabolism, as the current literature suggests that galactose cannot be consumed by *U. maydis* and is rather a toxic component inhibiting growth [21].

**Fig. 4:**
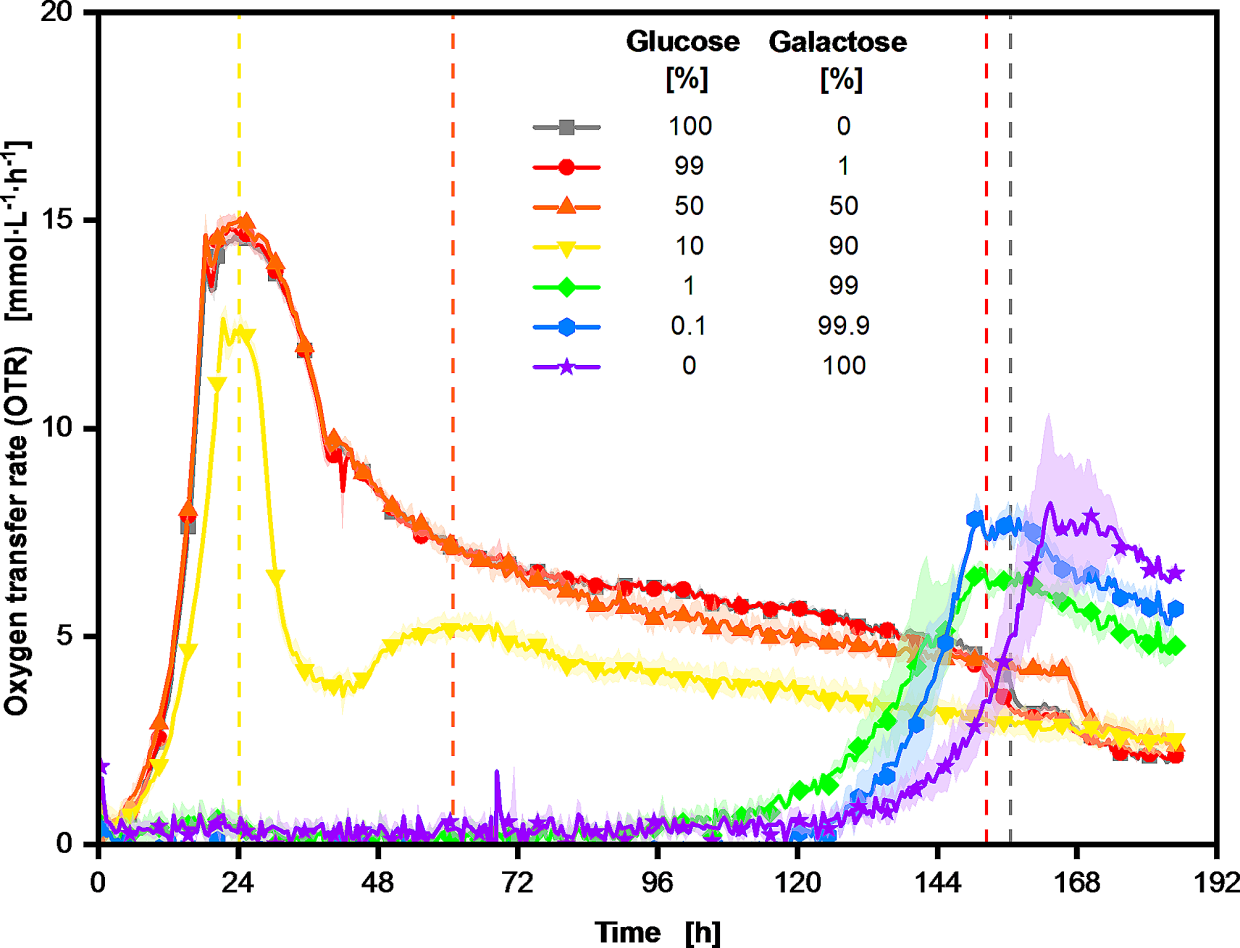
Comparison of the respiration activity of *U. maydis* MB215Δcyp1Δemt1 cultivations on different ratios between glucose and galactose. The Verduyn medium was supplemented with 100 g∙L^− 1^ glucose equivalent of the total carbon source, with reference to the respective percentage of glucose and galactose. Cultivations were conducted in biological duplicates, with the average values represented as a line and the min/max values illustrated as error shadows. Every fifth datapoint of the measurement is illustrated with a symbol. The vertical dashed lines represent the calculated time points of glucose consumption in the corresponding glucose-galactose mixture. The time points were calculated using the total oxygen consumption of the culture, which was determined using the OTR integral. For the lower glucose concentrations of 1 and 0.1 g∙L^− 1^, the time points of glucose depletion could not be determined due to the high noise at low OTR values. Carbon source consumption and additional recorded data is shown in the supplementary data S3. The pre culture was cultivated on 20 g∙L^− 1^ glucose

To gain deeper insights into the cultivation of *U. maydis* on galactose as sole carbon source, samples were taken at two process-relevant stages and microscopy was performed to study the morphology of the cells (Fig. 4).

As reference, a cultivation with glucose as carbon source was also sampled at identical cultivation stages. The cultivation stages chosen included the beginning of the growth phase and the middle of the triglyceride production phase. By comparing the two carbon sources glucose and galactose, a decisive morphological difference can be recognized: With galactose as carbon source, small cell aggregates appeared on the surface of the culture broth at the beginning of the growth phase. These cell aggregates became increasingly visible during the exponential growth phase. In addition, turbidity of the culture broth was also observed, which implies partial suspension growth. The difference between the two cultivations becomes clear when compared directly. While the cultivation on glucose shows yeast-like cells with strictly separated cells, both in the growth phase (Fig. 4A, B) and in the triglyceride production phase (Fig. 4E, F), the cultivation on galactose shows elongated and partially aggregated cells in the growth phase (Fig. 4C, D). This trend is continuing in the oil production phase, where the formation of large aggregates and stress-induced filaments can be seen (Fig. 4G, H). The produced intracellular lipid bodies can be seen in the microscopic images on both glucose (Fig. 4E) and on galactose (Fig. 4G). Based on these observations we hypothesize that either the long lag phase or galactose itself induces morphological changes resulting in stress filaments. Similar findings were made for yeasts, like *S. cerevisiae*, where a carbon source-dependent filamentation was observed [48]. In *U. maydis* and in different yeasts, the filamentous growth is associated with metabolic changes [29, 49]. These changes could lead to previously toxic metabolites having a less inhibitory effect or other metabolic pathways being used that do not generate toxic intermediates. For example, a paper by Andrews et al. from 2004 documented that many genes are subject to different regulatory mechanisms depending on whether *U. maydis* grows filamentous or yeast-like. Some of these genes could be assigned to the class of oxidoreductases, enzymes that among other reactions catalyze parts of the alternative pathway of galactose metabolism, the oxidoreductive pathway [29]. This hypothesis is supported by the fact that *U. maydis* already possesses the genetic equipment for a functional oxidoreductive galactose degradation pathway and that many filamentous growing fungi can also metabolize galactose [21, 22, 50]. In addition, the improved adaptability due to morphological changes has often been observed, especially in pathogenic species of fungi [51, 52]. In this context, fungi like *U. maydis* and other plant pathogens have also frequently been studied [52]. However, this morphological change in the organism does not appear to have any major impact on the assessment and optimization of oil production in *U. maydis*, since, as can be seen in Fig. 3, the oil yield is almost identical to that on glucose.

### Assessing the influence of glucose-galactose mixtures on the growth and oil production

**Fig. 5:**
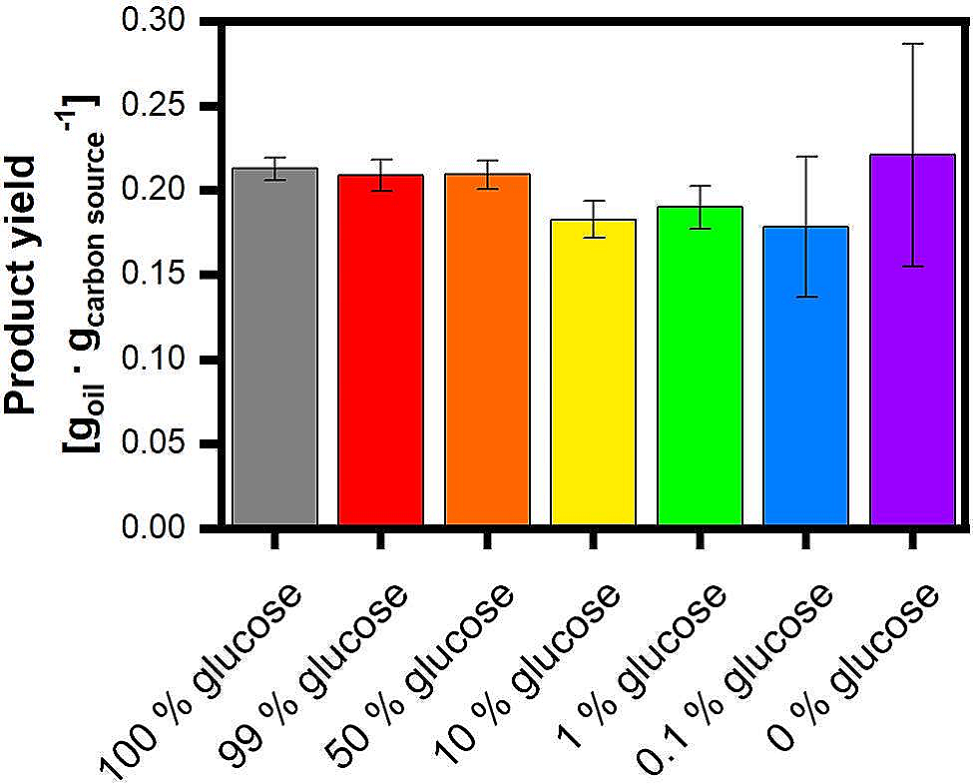
Carbon yields of triglycerides produced by *U. maydis* MB215Δcyp1Δemt1 on different mixtures between glucose and galactose (relating to Fig. 4). Extraction was performed in biological replicates, with the average values represented in the column chart and the min/max values illustrated as error bar. The Product yield was calculated by dividing the oil concentration at the end of the cultivation by the used carbon concentration. Residual, non-consumed carbon source was determined by HPLC and included in the calculation by subtracting it from the initial used carbon source concentration. Carbon source consumption and additional recorded data is shown in the supplementary data S3

*Ustilago maydis* utilizes partially the same transporters to take up galactose and glucose [53]. Since glucose has a higher affinity to this transporter, the ratio between glucose and galactose is crucial for in vitro toxicity [53]. This is due to the fact that as long as glucose is present in the medium, galactose is not absorbed or metabolized, and therefore no toxic intermediates are formed. Consequently, the greater the biomass that can be formed in the presence of glucose, the more rapidly the adaptation to galactose should be able to take place. Of particular interest with regard to the use of plant-derived feedstocks are the ratios with excess glucose, up to a ratio of 50:50. Galactose is present in numerous natural feedstocks in varying concentrations. In plant-derived feedstocks, free galactose constitutes 1–6% of the total mass, with this range corresponding approximately to that of glucose. Consequently, glucose-galactose ratios in which galactose is present in excess are rarely found in plant-derived feedstocks. It should be noted that these ratios are only further shifted in favor of glucose if the plant biomass is hydrolyzed before use. Given the considerable influence that the ratio of glucose to galactose can exert on the culture, the following experiment investigated the influence of different glucose-galactose ratios on the cultivation and the extension on the lag phase. The respiratory activity of the cultures is presented in Fig. 5.

By comparing the oxygen transfer rate of the *U. maydis* cultivations with different glucose-galactose mixtures, the impact of the glucose concentration on the growth patterns and duration of the lag phase can be assessed. In all cultivations, the course of the OTR is similar to that of the cultivations illustrated in Fig. 1. Exponential growth can be observed, followed by the onset of nitrogen limitation. Then, the OTR slowly decreases until carbon source depletion or stop of the experiment. A comparison of the two cultivations on pure glucose and pure galactose again reveals a significant difference in the duration of the lag phase of around 120 h. The mixtures, on the other hand, show a different picture. A proportion of just 1% glucose in the medium shortens the start of the growth phase by around 24 h. A proportion of 10% reduces the initial lag phase to the level of pure glucose. There is still a small difference between the OTR courses of the cultivation with 10% and 50%, which indicates the early switch of the carbon source from glucose to galactose at around 24 h. This switch of the carbon source, which is indicated by the yellow vertical dashed line, is characterized by a sharp drop in the OTR, which then leads to a plateau formation at an OTR of 5 mmol. Nevertheless, the addition of just 10% glucose to the medium results in a reduction of the cultures lag phase of around 120 h. The higher glucose percentages 50 and 99 % confirm this behavior and follow the OTR course of pure glucose. Although a slight variation can be seen between the three cultivations after the calculated timepoint of carbon source switch, indicated by the orange vertical dashed line.

The results of this experiment make it possible to state that the otherwise long lag phase on galactose can be considerably shortened by adding small amounts of glucose. This confirms the results obtained from agar plate cultivations [21]. Similar behavior was also observed in other yeasts [30, 54]. Since the same transporter is used for glucose and galactose uptake [21] and glucose is preferred, the presence of glucose should lead to the cells first metabolizing the glucose and then being confronted with the potential toxicity of galactose at higher cell densities, which might lead to faster adaption, due to bed hedging of the culture. This adaptation to new cultivation conditions through population heterogeneity, called bed hedging, has been demonstrated for several organisms and different mechanisms [55], as well as for glucose and galactose metabolism in yeast systems [56].

**Fig. 6:**
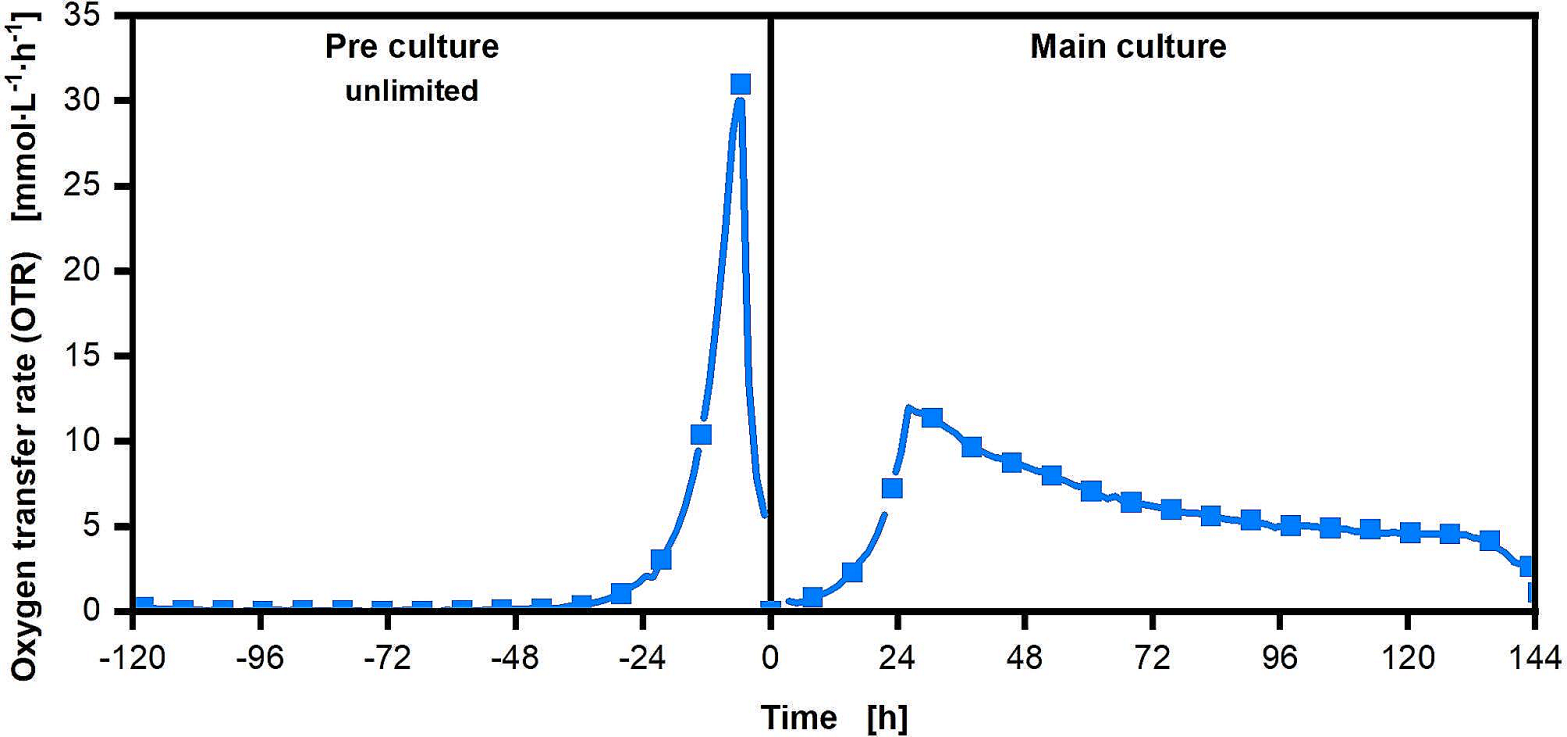
Respiration activity of a pre and main culture of *U. maydis* MB215Δcyp1Δemt1 on galactose as sole carbon source. Every fifth datapoint of the measurement is illustrated with a symbol. The Verduyn medium was supplemented with 20 g∙L-1 of galactose in the pre culture and with 100 g∙L-1 of galactose in the main culture. Pre culture was inoculated at an OD_600_ of 0.1 with a cryo-culture

To evaluate the influence of using carbon source mixtures on the triglyceride production of *U. maydis* the oil concentration was again determined in the respective crude cell extracts. Figure 6 illustrates the calculated triglyceride yield per consumed carbon, considering both triglyceride concentrations and the amount of consumed glucose and galactose.

Figure 6 confirms the systematic that could already be seen in Fig. 3. All the glucose-galactose-mixtures show a product yield of 0.18 to 0.22 g_triglycerides_∙g_carbon source_^−1^. Because of the short oil production phases in the cultivations of 1% glucose and lower, the error of the product yield increases, because the oil concentrations substantially decreases. These findings confirm the similar product yield on glucose and galactose, which was already evident in the carbon source screening (Fig. 1) and agreed well with other organisms found in literature [41, 42]. To summarize, with a glucose proportion of 10% it wa already possible to prevent the occurrence of an extended lag phase in the cultivation of *U. maydis* with galactose as main carbon source resulting in a behavior comparable to growth on glucose and only small influences on the measurable activity of the culture. A comparison of naturally occurring galactose-glucose ratios shows that high galactose content in regard to plant biomass is the exception rather than the rule. For this reason, it can be summarized that the galactose content in plant biomass should not strongly influence the growth of *U. maydis* in production processes.

### Adaption of *U. maydis* on galactose containing medium

In a second approach to shorten the lag-phase and investigate the adaption of *U. maydis* on medium containing galactose, the conventional pre culture on glucose was omitted. Instead, the pre culture was cultivated on 20 g∙L^− 1^ galactose. The OTR data of the pre culture on galactose as well as the main culture on galactose are shown in Fig. 2.

Interestingly, a slightly shorter lag phase in the pre culture was observed compared to the previously discussed main cultures, likely due to the reduced galactose concentration used for the pre culture. The pre culture was stopped shortly after galactose depletion indicated by the abrupt drop in the OTR after a cultivation time of 120 h. The cells were washed with 9 g∙L^− 1^ sodium chloride solution prior to inoculation of the main culture. The respiratory activity of the main culture showed a particularly interesting behavior. Although the main culture contains 100 g∙L^− 1^ galactose as sole carbon source, the culture does not show any lag phase at all but starts the exponential growth phase immediately after inoculation with a maximal growth rate of 0.146 h^− 1^. This growth rate is comparable to the other cultivations on pure galactose shown Figs. 1 and 5. This remarkable behavior of the culture represents an adaptation of the pre culture cells to galactose as a carbon source. As in the other experiments presented here (Figs. 1 and 5), the exponential growth phase continues until the nitrogen source is depleted. The formation of a single peak followed by a slowly decreasing respiratory activity can be observed. After the time of nitrogen depletion, the triglyceride production of the organism starts until galactose depletion. With a product yield of 0.19 g_triglycerides_∙g_carbon source_^−1^ this cultivation is in the same range as the previous cultivations on pure galactose, glucose, or the glucose-galactose mixtures. Another unique point compared to the other experiments presented here is the complete metabolization of the galactose and the successful conversion into oil. No Galactose was measured by HPLC in the end of the experiment and a corresponding 19.1 g∙L^− 1^ of oil could be extracted from the fully metabolized 100 g∙L^− 1^ of added galactose. This method also shows that the long lag phase can be prevented by an adapted cultivation protocol. The prolonged pre culture must be considered, but if cultivation in a fermenter is assumed, it may be worthwhile to rely on a slow pre-cultivation and a faster main-cultivation, as a fast-growing main culture reduces the risk of contamination in large-volume fermenter. An adaptation to a specific substrate during pre-cultivation has already been demonstrated for other organisms and substrates [57, 58]. However, when changing the primary carbon source in the main culture again, or when changing to a mixture of different carbon sources, the adaptation of the pre culture to galactose can lead to a slight prolongation of the lag phase in the main culture. The respiratory activity of a culture when changing from a galactose pre culture to a main culture with a mixture of carbon sources is shown in Figure S5. Here the lag phase is extended by approximately 2 h. However, if there is a high load of galactose in the mixture, this method could still lead to a shortening of the lag phase in the main culture.

## Conclusion and outlook

The investigation of the metabolic capabilities of *U. maydis* has revealed noteworthy findings that expand our understanding of its growth and product synthesis on various carbon sources, with a particular focus on galactose. In the presented experiments, *U. maydis* showed growth on all tested soluble saccharide building blocks of corn stover, consistent with expectations for a maize pathogen [59]. Interestingly, we observed that *U. maydis* exhibits growth on galactose, albeit with an extended lag phase of approximately 100 h. This challenges the previous hypothesis, that galactose degradation potentially leads to accumulation of toxic intermediates published by Schuler et al. in 2017 [21]. The observed growth on galactose could be connected to a differential regulation of genes for the two distinct galactose metabolization pathways in *U. maydis*, that were also described in the same publication [21]. Our microscopy observation of *U. maydis* cells during cultivation suggests that distinct growth patterns emerge in response to different carbon sources, implying a potential relationship between filamentous growth and galactose consumption. The literature also reports differences in gene expression between suspension and filamentous growth in *U. maydis*. In accordance with our findings, an increased activity of motifs, which are important for the oxidoreductive degradation pathway for galactose, was observed [29]. Additionally, we hypothesize that the expression of genes associated with the oxidoreductive pathway may be triggered in the presence of galactose, further supporting this connection.

Our study demonstrated that triglyceride production in *U. maydis* was achieved under nitrogen-limited conditions for all tested carbon sources. While glucose remains the preferred substrate for optimal product yield, galactose demonstrated a comparable yield, despite the prolonged lag phase. Furthermore, other saccharide building blocks of corn stover (fructose, sucrose, xylose and arabinose) demonstrated only slightly reduced yields. These results are promising with regard to the use of complex feedstocks as the basis for a future production process.

Additionally, we successfully implemented two methods to mitigate the extended lag phase on galactose. The use of glucose-galactose mixtures demonstrated a major reduction of the lag phase starting from glucose proportions smaller than 10%. Considering the scarcity of feedstocks with such high galactose to glucose ratios, it is possible to use most galactose-containing feedstocks for oil production with *U. maydis*. In case of pure galactose or abundance of glucose, the second tested method could be applied, to avoid the negative effects of galactose on the cultivation time. A pre culture grown on galactose eliminated the lag phase, indicating cellular adaptation to galactose as a carbon source, without impending triglyceride formation. By applying these two methods, substrates with high galactose content, such as crop or dairy waste streams, should be applicable for triglyceride production. This again demonstrates that *U. maydis* is a highly promising microorganism for converting biomass into microbial triglycerides.

### Electronic supplementary material

Below is the link to the electronic supplementary material.

**Supplementary Material 1**: **Figure S2:** Illustration of the respiration activity of *U. maydis* MB215Δcyp1Δemt1 on galactose as sole carbon source. OTR data also visible in Fig. 1. The Verduyn medium was supplemented with 100 g∙L^− 1^ galactose. Cultivation was conducted in duplicate, with the average values represented as a line and the min/max values illustrated as error shadows. Carbon source consumption and additional recorded data are shown in the supplementary data S1. **Figure S3:** Natural logarithm of the oxygen transfer rate (OTR) against the time of the exponential growth phase of *U. maydis* MB215Δcyp1Δemt1 on different carbon sources. The time was set to zero for all cultivations at the beginning of the exponential growth phase. The slope of this plot corresponds to the growth rate of the respective cultivation on the corresponding substrate [39]. **Figure S5:** Illustration of the respiration activity of *U. maydis* MB215Δcyp1Δemt1 on galactose and on a mixture of the carbon sources glucose, xylose, galactose, arabinose and sucrose. OTR data (black) also visible in Fig. 2 (main culture). The Verduyn medium was supplemented with 100 g∙L^− 1^ galactose or 100 g∙L^− 1^ glucose equivalent of the mixture with the same proportion of all carbon sources.

**Supplementary Material 2**: **Table S1**: Additional data to the carbon source screening with *U. maydis* MB215Δcyp1Δemt1. Growth rates were calculated by determining the slope of the natural logarithm of the oxygen transfer rate in the exponential growth phase of the respective cultivation. A graphical representation of this is illustrated in Figure S2. Product yield was calculated by dividing the oil concentration at the end of the cultivation by the used carbon concentration (Glucose normed start concentration minus final carbon source concentration). The final carbon concentration was determined at the end of cultivation by HPLC measurement. This corresponds to the following times for the respective carbon source used: glucose, fructose, sucrose = 144 h; arabinose, xylose and galactose = 221 h. Optical density was measured at 600 nm. Respiratory activity data is shown in Fig. 1, triglyceride production yield is presented in Fig. 3. **Table S4**: Additional data to the cultivation *U. maydis* MB215Δcyp1Δemt1 on different glucose-galactose ratios. Growth rates were calculated by determining the slope of the natural logarithm of the oxygen transfer rate in the exponential growth phase of the respective cultivation. Product yield was calculated by dividing the oil concentration at the end of the cultivation by the used carbon concentration (Start concentration minus final carbon source concentration). The final carbon concentration was determined at the end of cultivation by HPLC measurement. This corresponds to the following times for the respective carbon source used: glucose, fructose, sucrose = 144 h; arabinose, xylose and galactose = 221 h. Optical density was measured at 600 nm. Respiratory activity data is shown in Fig. 5, triglyceride production yield is presented in Fig. 6.

## Data Availability

The datasets used and/or analyzed during the current study are available from the corresponding author on reasonable request.
