# Supplementary material for: Studying microbial triglyceride production from corn stover saccharides unveils insights into the galactose metabolism of Ustilago maydis
Source: Microb Cell Fact. 2024 Jul 20;23:204. doi: 10.1186/s12934-024-02483-1 (PMC11264902; doi:10.1186/s12934-024-02483-1)
Supplement: Supplementary file 1 — Supplementary Material 1: Figure S2: Illustration of the respiration activity of U. maydis MB215Δcyp1Δemt1 on galactose as sole carbon source. OTR data also visible in Fig. 1. The Verduyn medium was supplemented with 100 g∙L− 1 galactose. Cultivation was conducted in duplicate, with the average values represented as a line and the min/max values illustrated as error shadows. Carbon source consumption and additional recorded data are shown in the supplementary data S1. Figure S3: Natural logarithm of the oxygen transfer rate (OTR) against the time of the exponential growth phase of U. maydis MB215Δcyp1Δemt1 on different carbon sources. The time was set to zero for all cultivations at the beginning of the exponential growth phase. The slope of this plot corresponds to the growth rate of the respective cultivation on the corresponding substrate [39]. Figure S5: Illustration of the respiration activity of U. maydis MB215Δcyp1Δemt1 on galactose and on a mixture of the carbon sources glucose, xylose, galactose, arabinose and sucrose. OTR data (black) also visible in Fig. 2 (main culture). The Verduyn medium was supplemented with 100 g∙L− 1 galactose or 100 g∙L− 1 glucose equivalent of the mixture with the same proportion of all carbon sources. [file 12934_2024_2483_MOESM1_ESM.pdf]

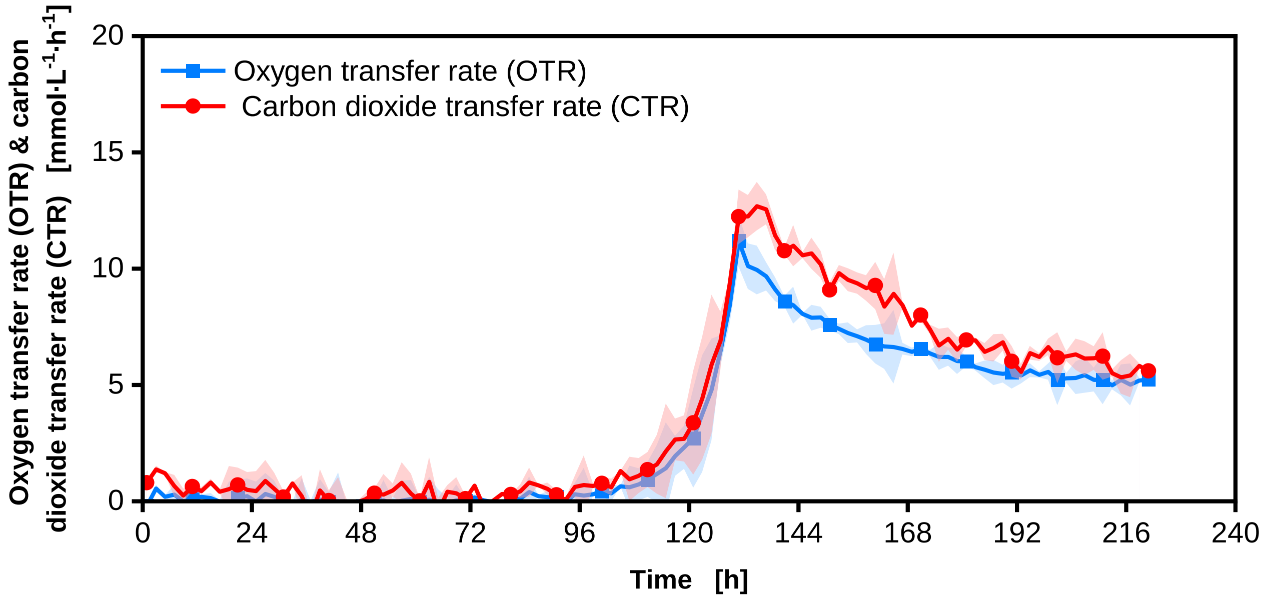

Figure S2: Illustration of the respiration activity of *U. maydis* MB215Δcyp1Δemt1 on galactose as sole carbon source. OTR data also visible in Figure 1. The Verduyn medium was supplemented with 100 g·L^{-1} galactose. Cultivation was conducted in duplicate, with the average values represented as a line and the min/max values illustrated as error shadows. Carbon source consumption and additional recorded data are shown in the supplementary data S1.

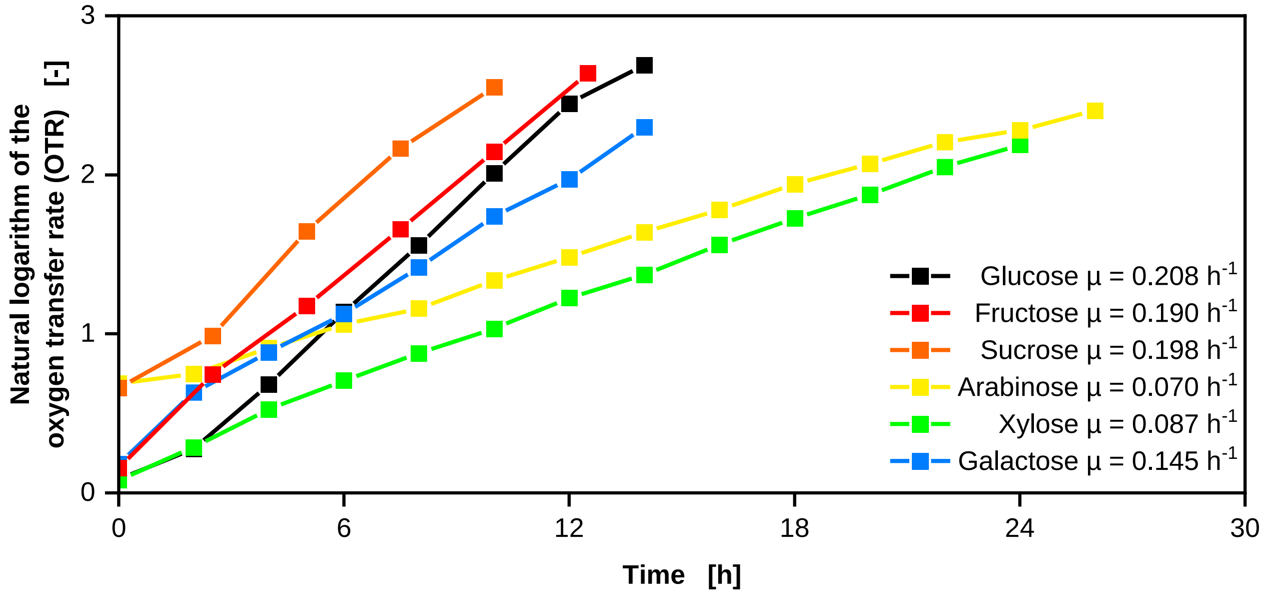

Figure S3: Natural logarithm of the oxygen transfer rate (OTR) against the time of the exponential growth phase of U. maydis MB215Δcyp1Δemt1 on different carbon sources. The time was set to zero for all cultivations at the beginning of the exponential growth phase. The slope of this plot corresponds to the growth rate of the respective cultivation on the corresponding substrate [56].

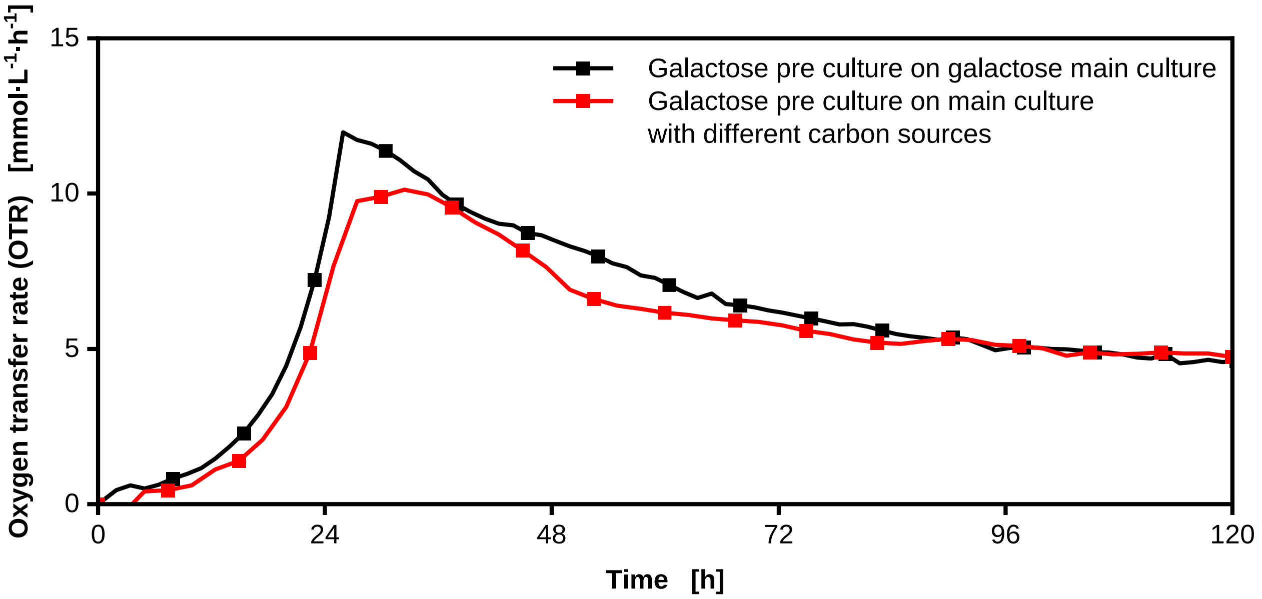

Figure S5: Illustration of the respiration activity of *U. maydis* MB215Δcyp1Δemt1 on galactose and on a mixture of the carbon sources glucose, xylose, galactose, arabinose and sucrose. OTR data (black) also visible in Figure 6 (main culture). The Verduyn medium was supplemented with 100 g·L^{-1} galactose or 100 g·L^{-1} glucose equivalent of the mixture with the same proportion of all carbon sources.
